# Upregulation of *TFDP1* and *CDC27* Plays an Important Role in Bronchiectasis

**DOI:** 10.1155/carj/2891024

**Published:** 2025-12-25

**Authors:** Kang-Kang Hong, Guo-Sheng Li, Rong-Quan He, Zhi-Guang Huang, Yi-Zhi Feng, Jin-Liang Kong, Lao-Dong Li

**Affiliations:** ^1^ Department of Geriatric Medicine, The Fourth Affiliated Hospital of Guangxi Medical University, LiuZhou, Guangxi, China, gxmu.edu.cn; ^2^ Ward of Pulmonary and Critical Care Medicine, The First Affiliated Hospital of Guangxi Medical University, Nanning, Guangxi, China, gxmu.edu.cn; ^3^ Department of Cardiothoracic Surgery, The First Affiliated Hospital of Guangxi Medical University, Nanning, Guangxi, China, gxmu.edu.cn; ^4^ Department of Medical Oncology, The First Affiliated Hospital of Guangxi Medical University, Nanning, Guangxi, China, gxmu.edu.cn; ^5^ Department of Pathology, The First Affiliated Hospital of Guangxi Medical University, Nanning, Guangxi, China, gxmu.edu.cn; ^6^ Ward of Pulmonary and Critical Care Medicine, The Fourth Affiliated Hospital of Guangxi Medical University, Liuzhou, Guangxi, China, gxmu.edu.cn

**Keywords:** biomarker, bronchiectasis, *CDC27*, cell cycle, *TFDP1*

## Abstract

**Background:**

Noncystic fibrosis (non‐CF) bronchiectasis is a chronic respiratory disease characterized by irreversible bronchial dilation, with an increasing global prevalence and substantial clinical burden. Despite the advances in symptomatic management, the underlying molecular mechanisms remain poorly understood. Transcription factor DP‐1 (*TFDP1*) and cell division cycle protein 27 (*CDC27*), which are implicated in tumorigenesis and cell cycle regulation, have not been explored in bronchiectasis.

**Methods:**

Gene expression data from the Gene Expression Omnibus dataset GSE97298 (27 patients with bronchiectasis vs. nine healthy controls) were analyzed to identify differentially expressed genes (DEGs) using edgeR (log_2_ fold‐change value ≥ 1.2, adj.*p* < 0.05). Protein–protein interaction (PPI) networks were constructed using STRING software. Clinical validation included 71 patients with bronchiectasis and 29 healthy controls. *TFDP1* and *CDC27* mRNA levels were quantified using real‐time polymerase chain reaction. Regulatory relationships were assessed using ChIP‐seq data (Cistrome DB), and pathway enrichment was performed using clusterProfiler to explore the potential molecular mechanisms of action of *TFDP1* and *CDC27* in non‐CF bronchiectasis.

**Results:**

In total, 355 DEGs were identified (45 upregulated and 310 downregulated genes). *TFDP1* and *CDC27* were significantly upregulated in patients with bronchiectasis (*p* < 0.01) and exhibited a strong positive correlation (*r* = 0.6104, *p* < 0.0001). ChIP‐seq confirmed *TFDP1* binding to the *CDC27* promoter region. Enrichment analysis revealed TFDP1‐associated DEGs involved in the cell cycle. *TFDP1* expression was correlated with the bronchiectasis severity index (*r* = 0.2904, *p* < 0.05).

**Conclusion:**

This study demonstrated the synergistic upregulation of *TFDP1* and *CDC27* in bronchiectasis and explored cell cycle regulation as an important potential mechanism by which *TFDP1* and *CDC27* contribute to the development of bronchiectasis. Therefore, *TFDP1* may serve as a biomarker of disease severity.

## 1. Introduction

Noncystic fibrosis bronchiectasis is a common chronic disease in adults. Clinical features include repeated symptoms of cough, sputum production, bronchial infection or hemoptysis, and permanent radiological dilation of the bronchi. Epidemiological studies have indicated that the prevalence of bronchiectasis continues to increase. Data from 2024 show that the adult prevalence in the United States has reached 714 cases/100,000, and the medical burden in Europe has increased significantly [1]. Asia has a large population base. However, large‐scale epidemiological studies on bronchiectasis are lacking. According to medical insurance data in China, the prevalence of adult bronchiectasis increased from 75.48 cases/100,000 in 2013 to 174.45 cases/100,000 in 2017, an increase of 2.31 times. From 2013 to 2017, the per capita total and hospitalization costs of patients with bronchiectasis increased by 2.18 and 1.83 times, respectively. The average annual number of hospitalizations in the 5 years was 1.20–1.24, and the prevalence rate of people over 40 years old exceeded 1200/100,000 [2]. A recent multicenter study (BE‐China) confirmed that compared with European and Indian cohorts, the clinical symptoms of Chinese patients with bronchiectasis are more severe, and the burden of this condition is exacerbated in low‐income areas. The number of patients with bronchiectasis is large, and clinical management needs to be standardized [3]. The treatment for patients with stable bronchiectasis includes airway clearance and expectorants. Antibiotic therapy is the primary treatment for patients with exacerbated bronchiectasis [4]. Repeated acute exacerbations of bronchiectasis may lead to antibiotic abuse, toxic side effects, increased risk of opportunistic infections, resistance to multiple antimicrobial agents, and considerable economic and life burdens on patients and society. The complex pathogenesis of bronchiectasis remains unclear. It is generally believed that various etiologies lead to impaired cell function in the bronchus, eventually leading to structural changes. Therefore, it is crucial to explore the molecular mechanisms and identify potential targets for the treatment and prevention of acute exacerbations of bronchiectasis.

The transcription factor (TF) DP‐1 (*TFDP1*) gene encodes a TF that is a member of the E2F family. It forms heterodimers with the E2F protein to enhance its DNA binding activity and promote the transcription of E2F target genes [5, 6]. This complex controls the transcriptional activity of many genes involved in cell cycle progression from the G1 to S phases [7]. The protein encoded by cell division cycle protein 27 (*CDC27*) is an important component of the anaphase‐promoting complex (APC) [8]. Its primary function is to regulate the cell cycle and mitotic processes [9, 10]. Several studies have reported that the expression of *TFDP1* is related to tumor proliferation [11, 12]. For example, in liver cancer, *TFDP1* overexpression promotes cell proliferation by upregulating cyclin E1 (CCNE1) [13]. In colon cancer, the direct binding of miR‐4711‐5p to *TFDP1* can result in the arrest of tumor cells in the G1 phase [14]. In lung adenocarcinoma, *TFDP1* is involved in the cell cycle, DNA replication, and other pathways and is associated with chemotherapeutic drug sensitivity, potentially serving as a prognostic marker [15]. Similarly, research on *CDC27* has focused on its role in tumors. It enhances the proliferation and invasion of tumor cells in gastric and colorectal cancers [16, 17]. In neuroblastoma, the CDC27–ODC1 axis accelerates tumor growth and induces iron death [18]. In nontumor diseases, *TFDP1* and E2F4 jointly regulate the proliferation of hematopoietic stem cells, with the deletion of *TFDP1* leading to the downregulation of 50% of cell cycle genes [19]. Additionally, *TFDP1* interacts with Kdm6b to regulate Trp53 expression, thereby affecting palatal development in mice [20]. A study on *CDC27* in systemic lupus erythematosus (SLE) showed that it was positively correlated with SLE disease activity and that inhibition of *CDC27* could slow down disease progression [21]. A study on idiopathic pulmonary fibrosis showed that hsa_circ_0044226 promotes EMT by upregulating *CDC27*, and its knockdown could inhibit *CDC27*, thus inhibiting the progression of pulmonary fibrosis [22]. However, the expression of *TFDP1* and *CDC27* in bronchiectasis and their relationship have not yet been reported. Therefore, this study aimed to investigate the expression and underlying molecular mechanisms of *TFDP1* and *CDC27* in bronchiectasis.

## 2. Materials and Methods

### 2.1. Collection of Datasets Consistent With Bronchiectasis

Access to multiple disease databases, including ArrayExpress, Sequence Read Archive, and Gene Expression Omnibus (GEO) datasets, is available online. By entering the search terms “Bronchiectasis AND *Homo sapiens*,” the chip dataset GSE97298 [23] from the GEO datasets was selected as suitable for the study. This dataset includes microarray data related to mRNA expression in 61 volunteers. Peripheral blood samples were collected before treatment. Among the 61 volunteers, 25 had nontuberculosis mycobacteria (NTM) infection with pulmonary disease manifesting as bronchiectasis or chronic obstructive pulmonary disease (COPD), 27 had non‐NTM infection, also with pulmonary disease manifesting as bronchiectasis or COPD, and the remaining nine were healthy individuals. Based on the clinical information provided by the author, which included lung function and lung computed tomography (CT) scores, and the objectives of this study, the mRNA expression data of 27 patients with complete clinical data and a confirmed diagnosis of bronchiectasis were selected as the bronchiectasis group, and the mRNA expression data of the nine healthy volunteers were selected as the control group. The inclusion and exclusion criteria are detailed in Table S1 .

### 2.2. Screening for Differentially Expressed Genes (DEGs)

The *R* language (v.4.1.0) edgeR package was used to preprocess the GEO data and identify DEGs. Log_2_ fold‐change (FC) value ≥ 1.2 and adj.*p* < 0.05 were applied together as the cutoff for upregulated DEGs screening. Log_2_ FC value ≤ 0.8 and adj. *p* < 0.05 were applied together as the cutoff for downregulated DEG screening.

### 2.3. Prediction of Potential TFs in Bronchiectasis

Differential gene expression during bronchiectasis may be regulated by several TFs. Binding analysis for regulation of transcription (BART) is a novel computational method and software package for predicting functional TFs associated with a genome spectrum [24]. To identify TFs in genes with significantly high expression, we used the TF prediction tool BART in *Python*.

### 2.4. Construction of the Protein–Protein Interaction (PPI) Network

The STRING database (https://string-db.org) was used to predict PPIs. This prediction is based on computational predictions, knowledge transfer between organisms, and data integration from other databases [25]. We used STRING to construct a PPI network of upregulated DEGs.

### 2.5. Expression of *TFDP1* and *CDC27* in the Bronchiectasis and Control Groups in the Chip Dataset GSE97298

The RNA‐Seq data were processed using Affymetrix Power Tools. Background correction was applied to probe intensities using the robust multiarray analysis algorithm, quantile normalization was performed, gene‐level expression was summarized from the intensities using the median polishing method, and the values were log_2_‐transformed.

### 2.6. Clinical Sample Collection and Real‐Time Polymerase Chain Reaction (PCR) Experiment

Peripheral blood samples were collected from patients with bronchiectasis (*n* = 71) and healthy controls (*n* = 29) in the Pulmonary and Critical Care Medicine Ward of the First Affiliated Hospital of Guangxi Medical University between April 2022 and April 2023, to verify the expression of *TFDP1* and *CDC27* using real‐time PCR. The inclusion and exclusion criteria were the same as those in Table S1. This study was approved by the Ethics Committee of the First Affiliated Hospital of Guangxi Medical University, China (Approval Number: 2023[E599‐01]), and informed consent was obtained from all participants. The privacy of the participants is strictly confidential. Age, body mass index (BMI), routine blood test results, and liver and kidney function of the volunteers were collected as baseline clinical data. In addition, clinical features such as smoking history, bronchiectasis severity score (E‐FACED score), bronchiectasis severity index (BSI), CT score (modified Reiff score), etiology, symptoms, signs, and pathogens were collected from the patients in the bronchiectasis group. Total RNA was isolated from peripheral blood using RNAiso Blood (Takara Bio, Tokyo, Japan), and complementary DNA (cDNA) was synthesized using a Reverse Transcription System Kit (PrimeScript™ RT Reagent Kit with gDNA Eraser, Tokyo, Japan), following the manufacturer’s instructions. Real‐time PCR was performed using TB Green Premix Ex Taq™ II (Takara Bio, Tokyo, Japan). Primers were designed by Sangon Biotech Co., Ltd. (Shanghai, China). The target genes included *TFDP1* and *CDC27*, with *β-actin* as an internal reference. The sequences and product sizes are listed in Table S2.

### 2.7. Regulatory Relationship Between *TFDP1* and Target Genes

The Cistrome Data Browser (Cistrome DB) contains ChIP‐seq, DNase‐seq, and ATAC‐seq data from humans and mice, including over 50,000 sample data of TFs, chromatin‐regulatory factors, and histone modifications. It is currently recognized as one of the most comprehensive ChIP‐seq databases [26, 27]. Therefore, we used the Cistrome DB to search for regulatory relationships between *TFDP1* and its target genes.

### 2.8. Pathway Enrichment Analysis of *TFDP1* and Its Related Upregulated DEGs

Based on the gene expression matrix, positive related genes (PRGs) of *TFDP1* were identified by Pearson’s correlation analysis in *R*, with *r* > 0.3 and *p* < 0.05. We then examined the intersection of the PRGs and upregulated DEGs. The resulting genes were subjected to Gene Ontology (GO) and Kyoto Encyclopedia of Genes and Genomes (KEGG) pathway analyses using the clusterProfiler package in *R*.

### 2.9. Statistical Analysis

The expression levels of *TFDP1* and *CDC27* in the microarray and clinical samples were log_2_‐transformed. SPSS 25.0 and *R* (v4.1.0) were used for statistical analysis and data processing, and GraphPad Prism software (version 8.0) was used to draw statistical graphs. For the comparison between two groups of continuous variables, if the data conformed to a normal distribution and the variance was homogeneous, an independent samples *t*‐test was used. If the data did not adhere to a normal distribution or the variance was uneven, the Mann–Whitney *U* test, a nonparametric test, was used. The chi‐square test was used to compare categorical variables between the two groups. Spearman’s or Pearson’s correlation tests were used to assess correlations. A receiver operating characteristic (ROC) curve was used to evaluate the expression patterns of DEGs in bronchiectasis.

## 3. Results

### 3.1. Identification of DEGs in Bronchiectasis

Based on the inclusion and exclusion criteria, 27 patients with a confirmed diagnosis of bronchiectasis were selected as the bronchiectasis group from the chip dataset GSE97298, and nine healthy volunteers were selected as the control group. The clinical information of both groups is presented in Table S3. There were no statistically significant differences in neutrophils, albumin, or C‐reactive protein levels between patients with bronchiectasis and the control group (*p* > 0.05).

**Figure 1:**
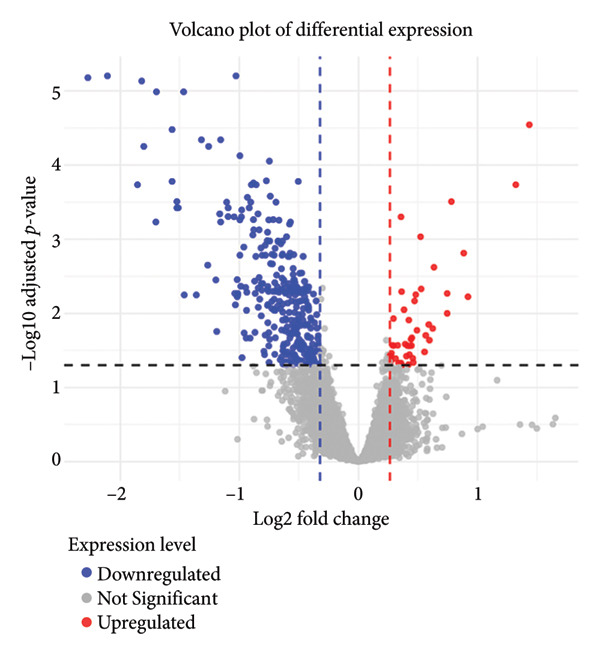
Volcano plots of DEGs.

DEGs were identified with a log_2_ FC value ≥ 1.2 and adj.*p* < 0.05 for upregulation and a log_2_ FC value ≤ 0.8 and adj.*p* < 0.05 for downregulation. Finally, 355 DEGs were identified, of which 45 were upregulated and 310 were downregulated. The volcano plots of the DEGs are presented in Figure 1.

### 3.2. Prediction of TFs for Upregulated DEGs

The BART algorithm was used to predict the TFs of the upregulated DEGs, and a potential TF, *TFDP1*, was identified.

### 3.3. PPI Network of Upregulated DEGs

**Figure 2:**
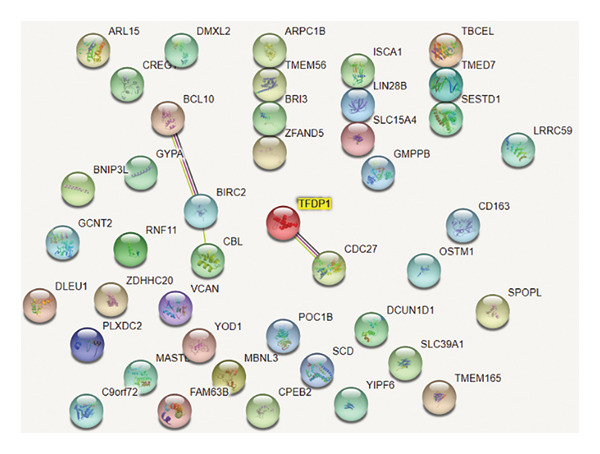
PPI network of upregulated DEGs.

The PPI network of the upregulated DEGs (Figure 2) showed that *TFDP1* is closely associated with *CDC27*.

### 3.4. Expression of *TFDP1* and *CDC27* in the Bronchiectasis and Control Groups in the Chip Dataset GSE97298

In the GSE97298 dataset, the expression levels of *TFDP1* and *CDC27* in the bronchiectasis group were significantly higher than those in the control group (Figures 3(a) and 3(b); *p* < 0.01). The correlation between *TFDP1* and *CDC27* expressions was investigated using Pearson’s correlation analysis. These results suggested a positive correlation between *TFDP1* and *CDC27* in the bronchiectasis group (Figure 3(c), *r* = 0.53, *p* < 0.05).

Figure 3The expression of *TFDP1* and *CDC27* in the bronchiectasis group and control group and the correlation between *TFDP1* and *CDC27* expressions in the chip dataset GSE97298. (a) Overexpression of *TFDP1* and *CDC27* in the bronchiectasis group; (b) ROC curve of *TFDP1* and *CDC27* in the bronchiectasis group and control group. ROC, receiver operating characteristic curve; (c) Expression correlation between *TFDP1* and *CDC27* expressions.

**Figure:**
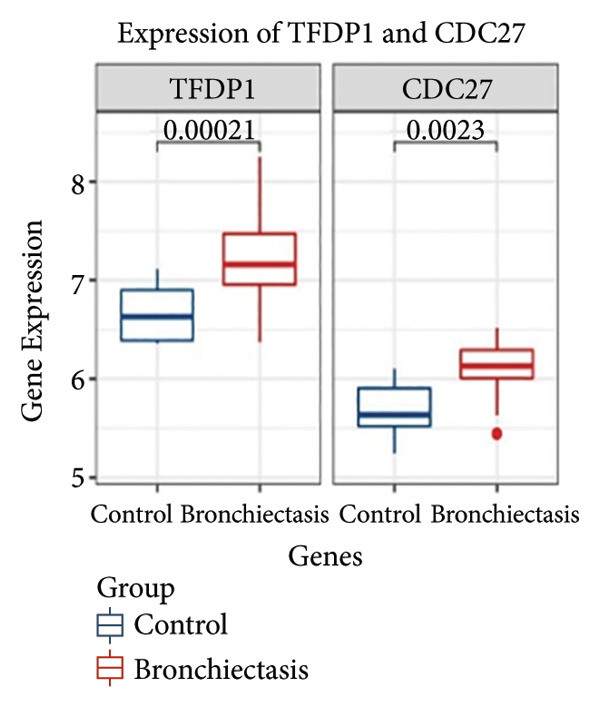
(a)

**Figure:**
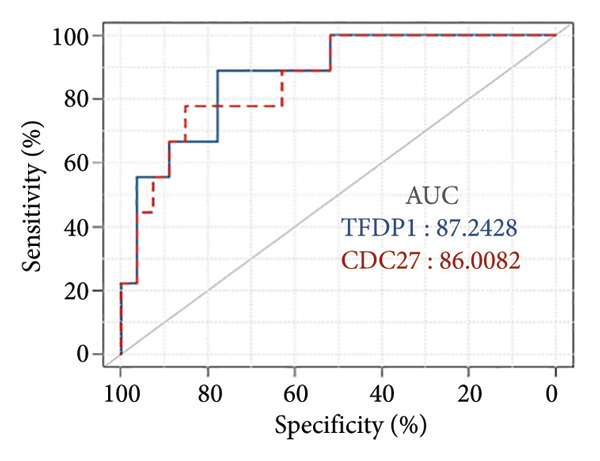
(b)

**Figure:**
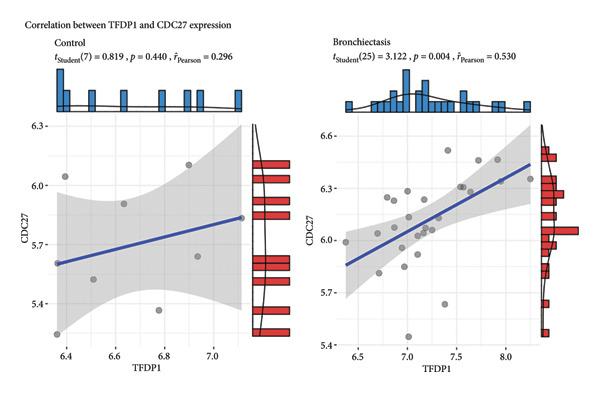
(c)

### 3.5. Expression of *TFDP1* and *CDC27* in Clinical Samples in the Bronchiectasis and Control Groups

To verify the expression of *TFDP1* and *CDC27* in bronchiectasis, we measured their expression in the peripheral blood of patients with bronchiectasis at the mRNA level. The clinical information of the bronchiectasis and control groups is presented in Table S4. As shown in Table S4, statistically significant differences were observed in BMI, percentages of neutrophils, lymphocytes, and monocytes, absolute values of neutrophils, lymphocytes, and monocytes, aspartate aminotransferase level, and albumin level between the bronchiectasis and control groups (*p* < 0.05). We found that the expression of *TFDP1* and *CDC27* in the bronchiectasis group was higher than that in the control group (*p* < 0.01; Figures 4(a), 4(b)).

Figure 4Expression of *TFDP1* and *CDC27* in the bronchiectasis and control groups and relationship between *TFDP1* and *CDC27*. (a) The expression of *TFDP1*; (b) the expression of *CDC27*;(c) correlation between *TFDP1* and *CDC27*; (d) expression of *CDC27* in high expression of *TFDP1* and low expression of *TFDP1* groups. Note: ∗∗*p* < 0.01; ∗∗∗∗*p* < 0.0001.

**Figure:**
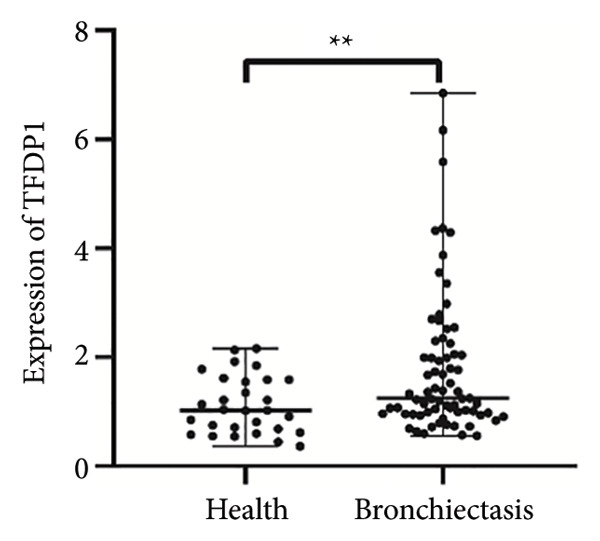
(a)

**Figure:**
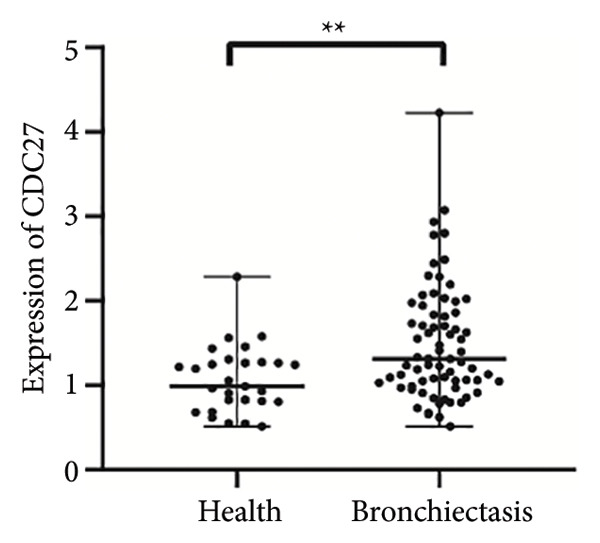
(b)

**Figure:**
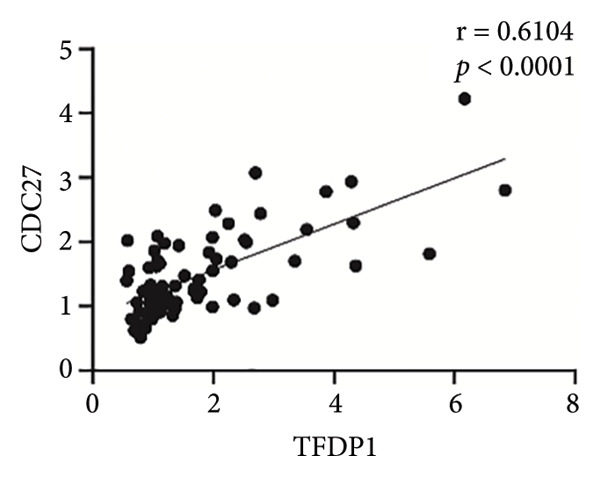
(c)

**Figure:**
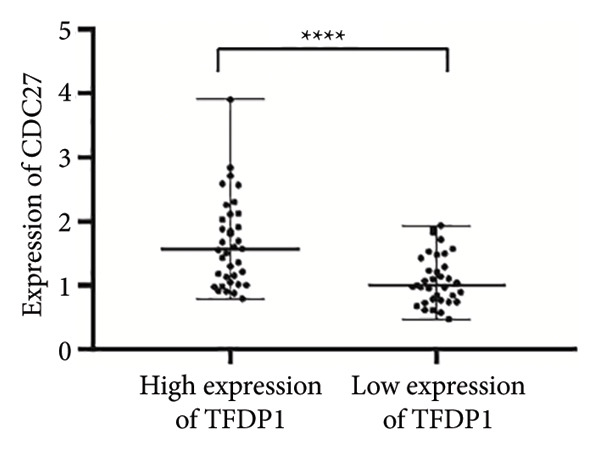
(d)

Pearson’s analysis indicated a positive correlation between *TFDP1* and *CDC27* expressions (*r* = 0.6104, *p* < 0.01; Figure 4(c)). To further investigate the relationship between *TFDP1* and *CDC27*, we divided 71 patients with bronchiectasis into two groups based on the median expression level of *TFDP1* (36 patients with high *TFDP1* expression and 35 patients with low *TFDP1* expression). The results showed that *CDC27* was also highly expressed in the high *TFDP1* expression group, and the differences were statistically significant (*p* < 0.0001; Figure 4(d)).

### 3.6. The Correlation Between TFDP1 and CDC27 and Other Clinical Indicators in Bronchiectasis

Concurrently, we analyzed the correlation between *TFDP1* and *CDC27* and other clinical indicators in bronchiectasis (Table 1). The results showed that *TFDP1* expression in bronchiectasis was positively correlated with the BSI (*r* = 0.2904, *p* < 0.05). *CDC27* expression level in bronchiectasis was negatively correlated with leukocyte (*r* = −0.3381, *p* < 0.05), neutrophil (*r* = −0.2768, *p* < 0.05), and absolute monocyte (*r* = −0.3259, *p* < 0.05) counts.

**Table 1:** Correlations between the expression levels of TFDP1, CDC27, and clinical indicators (n = 71).

Clinical indicators	TFDP1		CDC27	
	r	*p*	r	*p*
Age (years)	0.0632	0.6006	−0.8874	0.4618
BSI	**0.2904**	**0.0140**	0.0739	0.5404
E‐FACED	−0.0074	0.9509	−0.2222	0.0626
E‐Reiff	0.0180	0.8816	−0.0062	0.9593
Body mass index (kg/m^2^)	−0.0625	0.6045	0.08636	0.4739
Leukocyte (× 10^9^/L)	0.0177	0.8833	**−0.3381**	**0.0039**
Platelet (× 10^9^/L)	0.0246	0.8384	−0.2164	0.0699
Percentage of neutrophils (%)	0.1564	0.1928	−0.0732	0.5443
Percentage of lymphocyte (%)	−0.1543	0.1990	0.1080	0.3700
Percentage of eosinophils (%)	−0.0456	0.7057	−0.0450	0.7094
Percentage of basophils (%)	0.0751	0.5336	0.1601	0.1824
Percentage of monocytes (%)	−0.0557	0.6446	−0.1204	0.3174
Neutrophils (× 10^9^/L)	0.0880	0.4654	**−0.2768**	**0.0195**
Absolute value of lymphocytes (× 10^9^/L)	−0.1250	0.2988	−0.1401	0.2439
Absolute value of monocytes (× 10^9^/L)	−0.0235	0.8459	**−0.3259**	**0.0055**
Absolute value of eosinophils (× 10^9^/L)	0.0388	0.7480	−0.08744	0.4684
Absolute value of basophils (× 10^9^/L)	0.1600	0.1824	0.01194	0.9213
Aspartate aminotransferase (U/L)	0.0049	0.9680	−0.0631	0.6007
Alanine aminotransferase (U/L)	−0.0106	0.9304	0.06582	0.5855
Albumin (g/L)	−0.2026	0.0902	0.1298	0.2807
Glutamyl transferase(U/L)	0.0729	0.5455	0.1337	0.2662

*Note:* Bold values indicate statistical significance (*p* < 0.05).

### 3.7. Regulatory Relationship Between *TFDP1* and Target Genes

**Figure 5:**
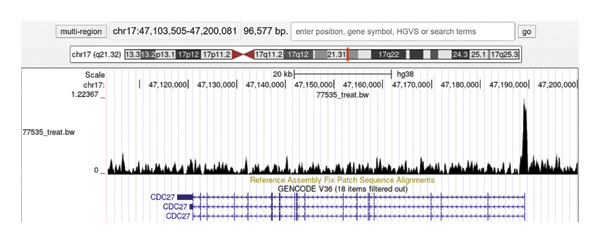
The potential regulatory relationship of *TFDP1* on *CDC27*.

To explore the relationship between *TFDP1* and *CDC27*, we used the Cistrome DB to query the presence of ChIP‐seq binding peaks with *TFDP1* in the promoter region of the *CDC27* transcription start site. This analysis suggested that *TFDP1* regulates the transcription of *CDC27* and affects its expression (Figure 5).

### 3.8. Pathway Enrichment Analysis of *TFDP1* and Its Related Upregulated DEGs

**Figure 6:**
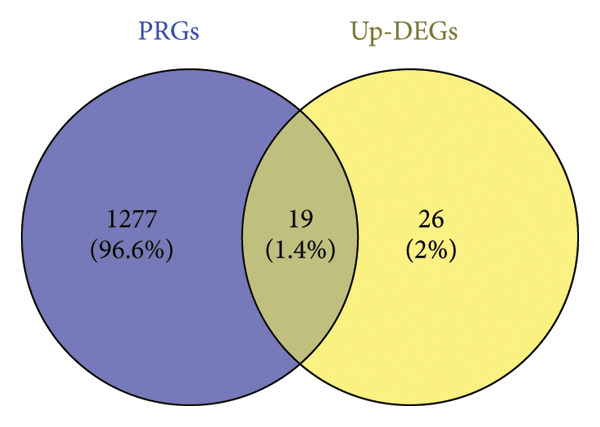
Selection of related upregulated DEGs of *TFDP1*.

**Figure 7:**
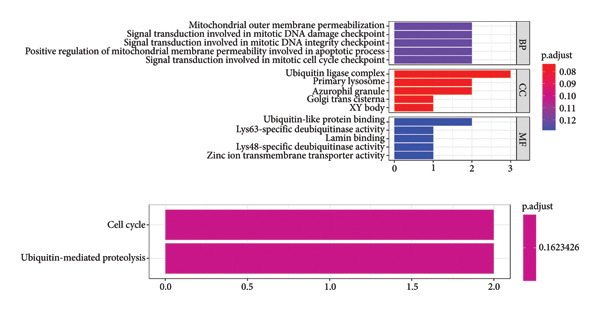
Enrichment analysis of *TFDP1* and its related upregulated DEGs.

Based on the gene expression matrix, 1277 PRGs associated with *TFDP1* were identified. These PRGs were intersected with 45 upregulated DEGs in the bronchiectasis group to obtain the *TFDP1*‐related upregulated DEGs (Figure 6). To explore the molecular mechanisms of *TFDP1* in bronchiectasis, we performed GO function and KEGG pathway analyses of the upregulated DEGs of *TFDP1*. GO analysis revealed that these genes clustered in GO terms related to mitochondria, mitosis, and ubiquitin, including “Signal transduction involved in mitotic DNA damage checkpoint” (biological processes), “ubiquitin ligase complex” (cell components), and “ubiquitin‐like protein binding” (molecular functions). In the KEGG analysis, these related upregulated DEGs of *TFDP1* were enriched in the “cell cycle” and “ubiquitin‐mediated proteolysis” pathways (Figure 7).

## 4. Discussion

The etiology of bronchiectasis is diverse, and the pathogenesis is very complex. Unlike cystic fibrosis, which is mainly caused by a single gene defect, non‐CF bronchiectasis does not have clear environmental exposure factors for the pathogenesis of COPD. This study focuses on the changes in the expression levels of *TFDP1* and *CDC27* in bronchiectasis and their clinical significance and explores the molecular mechanisms of their mutual regulation in the occurrence and development of bronchiectasis.

Cell cycle is a series of complex processes of cell content replication and division, involving a variety of regulatory factors. Any variation in these regulatory mechanisms can lead to gene mutations or chromosome number abnormalities, resulting in genomic instability. *TFDP1* and *CDC27* are both key genes regulating the cell cycle. *TFDP1* is involved in the regulation of apoptosis, cell cycle, and DNA damage repair by interacting with the E2F protein [28, 29]. *CDC27* is an important component of APC/C, which is involved in the regulation of cell cycle progression and chromosome segregation [8, 30]. The roles of *TFDP1* and *CDC27* in diseases were mostly concentrated in tumor‐related diseases, but the roles in bronchiectasis have not been reported. Our findings demonstrated that both chip datasets and clinical samples showed that *TFDP1* and *CDC27* were significantly overexpressed in peripheral blood samples of patients with bronchiectasis compared with healthy controls, with a strong positive correlation between their expression levels. High expression of *TFDP1* was often accompanied by high expression of *CDC27* in the bronchiectasis group. ChIP‐seq data further revealed a regulatory relationship between *TFDP1* and *CDC27*, indicating that *TFDP1* may directly modulate *CDC27* transcription. Pathway analysis highlighted the enrichment of *TFDP1*‐related upregulated DEGs in cell cycle and ubiquitin‐mediated proteolytic pathways, aligning with their established roles in cellular proliferation and protein degradation. Consistent with the results of the present study, *TFDP1* may play a major role in the cell cycle by regulating the transcription of *CDC27*. There was also no report on the transcriptional regulation relationship between *TFDP1* and *CDC27*. Our experimental results found that there may be such a relationship in bronchiectasis. Airway epithelium is the first barrier to resist infection and external environmental pressure. When epithelial cells are stimulated by pathogens or environmental changes, they may activate *TFDP1* and then transcriptionally regulate *CDC27*, affecting cell cycle pathways, resulting in the destruction of normal epithelial cell renewal, driving abnormal cell proliferation in bronchial tissues, and ultimately causing the destruction of bronchial wall structure and irreversible expansion. In addition, neutrophils, monocyte macrophages, and T lymphocytes as innate immune cells are involved in the development of bronchiectasis [31–33]. The increased expression of *TFDP1* and *CDC27* may affect the cell cycle of these immune cells and regulate the proliferation and death of immune cells. After the currently recognized treatment of bronchiectasis, such as antibiotics, airway clearance, immune regulation, and other treatments, how *TFDP1* and *CDC27* change and whether some drugs that affect the cell cycle can be used to treat bronchiectasis need further research.

Furthermore, in the study of the relationship between *TFDP1* and *CDC27* and clinical parameters, we observed a positive correlation between *TFDP1* expression and BSI, which was not previously reported, indicating that *TFDP1* can be used as a biomarker of disease severity. *CDC27* was negatively correlated with leukocytes, neutrophils, and monocytes, which also proved that *CDC27* participates in the activities of immune cells. However, the specific mechanism requires further investigation.

This study has several limitations. First, the reliance on peripheral blood samples rather than lung tissue limits the direct inference of local gene expression in the affected airways. Second, although changes in mRNA levels were validated, protein expression and post‐translational modifications were not assessed. Finally, a larger sample size is required for future studies. Future studies should include larger and more diverse samples to validate our results.

## 5. Conclusion

In summary, the current study revealed that *TFDP1* and *CDC27* were highly expressed in the bronchiectasis group and highlighted the potential regulatory effects of *TFDP1* on *CDC27*. Our study also explored cell cycle regulation, which may be an important mechanism by which *TFDP1* and *CDC27* participate in bronchiectasis development. The present study provides novel insights into the molecular mechanisms underlying bronchiectasis by identifying *TFDP1* and *CDC27* as key genes that are upregulated in this chronic respiratory disease.

## Ethics Statement

This study involves human participants and was approved by the Ethics Committee of the First Affiliated Hospital of Guangxi Medical University, China (Approval Number: 2023[E599‐01]). Participants gave informed consent to participate in the study before taking part.

## Consent

Written informed consent has been obtained from the patients to publish this paper.

## Disclosure

All authors have read and agreed to the published version of the manuscript.

## Conflicts of Interest

The authors declare no conflicts of interest.

## Author Contributions

Jin‐Liang Kong and Lao‐Dong Li participated in the research design. Kang‐Kang Hong and Yi‐Zhi Feng conducted experiments. Guo‐Sheng Li, Rong‐Quan He, and Zhi‐Guang Huang performed the data analysis. Kang‐Kang Hong, Guo‐Sheng Li, Jin‐Liang Kong, and Lao‐Dong Li wrote or contributed to the writing of the manuscript.

## Funding

This work was supported by the Liuzhou Science and Technology Planning Project (2024YB013A007), the Research Project Grant of Guangxi Health Committee (Z‐B20231379), and the Guangxi Medical and Health Key Discipline Construction Project.

## Supporting Information

Supporting Information 1. Table S1: Inclusion and exclusion criteria of the bronchiectasis group and the control group.

Supporting Information Table S2: Primer information list.

Supporting Information Table S3: Clinical information of the bronchiectasis group and the control group of donated blood samples.

## Supporting information


**Supporting Information** Additional supporting information can be found online in the Supporting Information section.

## Data Availability

Data are available upon reasonable request.
