# Supplementary material for: Upregulation of TFDP1 and CDC27 Plays an Important Role in Bronchiectasis
Source: Can Respir J. 2025 Dec 25;2025:2891024. doi: 10.1155/carj/2891024 (PMC12740773; doi:10.1155/carj/2891024)
Supplement: Supplementary file 1 — Supporting Information Additional supporting information can be found online in the Supporting Information section. [file CARJ-2025-2891024-s001.docx]

Table S1. Inclusion and exclusion criteria of bronchiectasis group and control group

| Group | Inclusion Criteria | Exclusion Criteria |
| --- | --- | --- |
| Bronchiectasis Group(n=27) | Age>18 years | Pregnancy |
|  | Clinical diagnosis of bronchiectasis | Taking Immunosuppressants |
|  | Before treatment | Malignant Tumor |
| Control Group(n=9) | Age>18 years | Pregnancy |
|  | Chest X-ray or lung CT is normal | Complicated with chronic lung disease |
|  | No infectious disease in recent month | Used drugs in recent one month |

Table S2. Primer information list

| Molecule | Primer sequences |
| --- | --- |
| TFDP1 | Forward:5’-AGCCTTCCGACTCCTCACCTTG-3’ |
|  | Reverse:5’-GCTCGTCTGCCACTTCGTTGTAG-3’ |
| CDC27 | Forward:5’-TCACAGAGTGGAAATAGCCGAGAGG-3’ |
|  | Reverse:5’-TTCTTCGAGGCAGTGCGTTTGG-3’ |
| β-actin | Forward:5’-CCTGGCACCCAGCACAAT-3’  Reverse:5’-GGGCCGGACTCGTCATAC-3’ |

Table S3. Clinical information of the bronchiectasis group and control groups of donated blood samples

(n=36)

| Clinical indicators | Bronchiectasis group (n=27) | Control group  (n=9) | | *P* |
| --- | --- | --- | --- | --- |
| Neutrophils (×10^9^/L) | 3.80 (3.30,4.60) | 3.30 (2.75,3.30) | | 0.349 |
| Albumin (g/L) | 40.00 (37.00,43.00) | 43.00 (40.00,44.00) | 0.180 | |
| C-reactive protein (mg/L) | 3.00 (1.00,7.00) | 1.00 (1.00,3.50) | 0.387 | |

Table S4. Clinical information of the bronchiectasis group and control groups of donated blood samples

(n=100)

| Clinical indicators | Bronchiectasis group (n=71) | Control group  (n=29) | | *P* |
| --- | --- | --- | --- | --- |
| Sex (male/female) | 32(45.07%)/39(55.93%) | 13(44.83%)/16(55.17%) | | 0.982 |
| Age (Years) | 57 (49,64) | 53 (43,65.5) | | 0.221 |
| Body mass index（kg/m^2^） | 20.03±0.40 | 22.13±0.33 | | 0.002 |
| Leukocyte (×10^9^/L) | 6.70 (5.98,8.38) | 6.17 (4.95,7.49) | | 0.058 |
| Platelet (×10^9^/L) | 277.2 (228.6,304) | 267.5 (240.95,302.8) | | 0.952 |
| Percentage of neutrophils (%) | 63.43±12.46 | 55.70±8.59 | | 0.003 |
| Percentage of lymphocyte (%) | 23.60 (17.65,33.10) | 32.00 (17.65,33.10) | | 0.001 |
| Percentage of eosinophils (%) | 1.98 (1.20,3.50) | 2.00 (1.60,3.15) | | 0.933 |
| Percentage of basophils (%) | 0.53 (0.30,0.65) | 0.60 (0.40,0.69) | | 0.348 |
| Percentage of monocytes (%) | 8.20 (6.59,9.54) | 5.70 (5.31,7.20) | | 0.001 |
| Neutrophils (×10^9^/L) | 4.29 (3.08,5.87) | 3.37 (2.69,4.36) | | 0.009 |
| Absolute value of lymphocytes (×10^9^/L) | 1.71±0.07 | 2.09±0.13 | | 0.019 |
| Absolute value of monocytes (×10^9^/L) | 0.54 (0.46,0.66) | 0.38 (0.31,0.48) | | 0.001 |
| Absolute value of eosinophils  (×10^9^/L) | 0.15 (0.07,0.26) | 0.12 (0.09,0.24) | | 0.59 |
| Absolute value of basophils  (×10^9^/L) | 0.03 (0.02,0.05) | 0.03 (0.02,0.04) | | 0.644 |
| Aspartate aminotransferase  (U/L) | 22.00 (17.00,23.00) | 17.00 (14.50,23.00) | | 0.008 |
| Alanine aminotransferase  (U/L) | 14.00 (10.00,20.00) | 14.00 (10.00,23.00) | | 0.906 |
| Albumin (g/L) | 38.59±0.62 | 45.97±0.36 | 0.001 | |
| Glutamyl transferase (U/L) | 25.00 (15.00,40.00) | 23.00 (15.00,41.50) | 0.979 | |
